# Evolution of Adaptive Variation in the Mosquito *Culex pipiens*: Multiple Independent Origins of Insecticide Resistance Mutations

**DOI:** 10.3390/insects12080676

**Published:** 2021-07-27

**Authors:** Valentina Mastrantonio, Daniele Porretta, Valentina Lucchesi, Nurper Güz, Naciye Sena Çağatay, Romeo Bellini, John Vontas, Sandra Urbanelli

**Affiliations:** 1 Department of Environmental Biology, Faculty of Mathematical, Physical and Natural Sciences, Sapienza University of Rome, 00185 Rome, Italy; valentina.mastrantonio@uniroma1.it (V.M.); valentina.lucchesi@uniroma1.it (V.L.); sandra.urbanelli@uniroma1.it (S.U.); 2 Molecular Entomology Laboratory, Department of Plant Protection, Faculty of Agriculture, Ankara University, Ankara 06100, Turkey; nurperguz@agri.ankara.edu.tr (N.G.); nsenacagatay@hotmail.com (N.S.Ç.); 3 Medical and Veterinary Entomology Department, Centro Agricoltura Ambiente “G. Nicoli”, 40014 Bologna, Italy; rbellini@caa.it; 4 Department of Crop Science, Pesticide Science Lab, Faculty of Crop Science, Agricultural University of Athens, 11855 Athens, Greece; vontas@imbb.forth.gr; 5 Institute of Molecular Biology and Biotechnology, Foundation for Research and Technology Hellas, 70013 Heraklion, Crete, Greece

**Keywords:** insecticide resistance, vector control, insect growth regulators, Chitin synthase

## Abstract

**Simple Summary:**

The application of insecticides to control insect pests can result in the evolution of resistance. Within a population, the individuals carrying the resistant mutation survive after insecticide application while the others die, leading to the spread of resistance. The finding that the same mutations confer insecticide resistance in different species or populations raises the question how often these mutations arise in natural populations. Resistant mutations can originate once within a population and then spread. Alternatively, multiple origins can occur within the same population or in different geographic areas. Here, we used a phylogenetic approach to investigate the origin of three mutations conferring resistance to diflubenzuron insecticide in Italian and Turkish populations of the mosquito *Culex pipiens*. Our results support a scenario of multiple origins of the mutations. Resistance is a major threat to mosquito control, and these findings help inform resistance management. At the same time, insecticide resistance is an informative model for studying the origin of adaptive variation. In the words of Rachel Carson, “If Darwin were alive today, he would be astounded and delighted by the impressive verification that his theories of the survival of the fittest are receiving from the insect world” (Silent Spring, 1962)

**Abstract:**

Insecticide resistance is an informative model for studying the appearance of adaptive traits. Simultaneously, understanding how many times resistance mutations originate is essential to design effective resistance management. In the mosquito *Culex pipiens*, target–site resistance to the insecticide diflubenzuron (DFB) has been recently found in Italian and Turkish populations. Three point mutations confer it at the codon 1043 of the chitin synthase 1 gene (*chs-1*): I1043L, I1043M, and I1043F. Whether the resistant mutations originated independently from different susceptible alleles or sequentially from resistant alleles and whether resistant alleles from Italy and Turkey have originated once or multiple times remain unresolved. Here, we sequenced a fragment of the *chs-1* gene carrying the resistant mutations and inferred the phylogenetic relationships among susceptible and resistant alleles. Confirming previous findings, we found the three mutations in Italy and the I1043M in Turkey. Notably, the I1043F was also found for the first time in Turkish samples, highlighting the need for extensive monitoring activities. Phylogenetic analyses are consistent with an independent origin of the I1043F, I1043M, and I1043L mutations from different susceptible alleles and with multiple independent origins of the Italian and Turkish I1043M and I1043F alleles.

## 1. Introduction

The understanding of how new beneficial traits originate is still a major goal in evolutionary biology. In this context, insecticide resistance has proved to be an informative model for studying the acquisition of new adaptive traits. The molecular basis of insecticide resistance to almost classes of insecticides has been indeed elucidated and our ability to detect resistant mutations has significantly improved in recent years [1,2,3,4,5].

The number of times different mutations have appeared is a major issue in understanding the adaptive traits’ appearance. This subject can be addressed by surveying the phylogenies of extant susceptible and resistant alleles. If the same mutation has occurred independently more than once, then we should be able to see the susceptible progenitor allele for each resistance allele that may still carry similar flanking sequences [1].

In this paper, we investigated the origin of the insecticide resistance to diflubenzuron (DFB) in the mosquito *Culex pipiens*. DFB is a Chitin Insect Inhibitor (CSI) widely used to control insect pests in agriculture as well as vectors in public health [6,7]. Its mechanism of action involves the interaction with the chitin synthase 1 (CHS1), the enzyme responsible for chitin synthesis in the cuticle [8,9]. DFB mainly acts by inhibiting the chitin synthesis and deposition, causing abortive molting and other insect physiology alterations [8].

In *Cx. pipiens*, DFB resistance is associated with three point mutations in the chitin synthase 1 gene (*chs-1*) at the amino codon 1043. The susceptible individuals carry the “ATC” gene sequence corresponding to the Isoleucine amino acid (I1043). In contrast, the mutant individuals carry “CTC,” “ATG,” or “TTC” sequences corresponding to the Leucine (I1043L), Methionine (I1043M), or Phenylalanine amino acids (I1043F), respectively [9,10]. The three mutations have been functionally validated with CRIPSR/Cas9 in *D. melanogaster*, and it was shown that they confer significant levels of resistance: the I1043M and the I1043F mutations resulted in a Resistance Ratio of >15,000 fold and the I1043L mutation in a Resistance Ratio of >20 fold [8,9]. Furthermore, quality control treatment, regularly conducted in the frame of the Emilia-Romagna (Northern Italy) mosquito control plan, showed that resistance can dramatically affect DFB performance in the field [10].

Since the first detection of DFB resistance in 2015 in Italy, *Cx. pipiens* populations from Portugal, France, Spain, Italy, Greece, Turkey, and Israel were screened for the occurrence of resistant mutations [9,10,11,12]. Except for two heterozygous individuals, i.e., I1043/I1043F recently found in Montpellier (France) [10], Italy and Turkey are the major areas where the resistant mutations occur. In particular, all three resistant alleles were found in the Emilia-Romagna region (Italy), with the I1043L and I1043M mutations having a frequency up to 60% and 77%, respectively, and the I1043F mutation a frequency <10% [10,11]. In the Mugla province (Turkey), the I1043L and I1043M DFB mutations were reported with a frequency of 28.1% and 43.3%, respectively [12].

To date, the origin of these mutations remains unresolved. Here, we aimed to assess whether: (i) the I1043F, I1043M and I1043L mutations conferring resistance have originated independently from different susceptible alleles or whether they come from mutations in resistant alleles; (ii) the resistant alleles found in Italy and Turkey have originated from multiple independent mutation events. To this aim, we sequenced a fragment of the *chs-1* gene, which carries the I1043-resistant mutations, and surveyed the phylogenies of susceptible and resistant alleles.

## 2. Materials and Methods

### 2.1. Study Sites and Samples 

**Figure 1:**
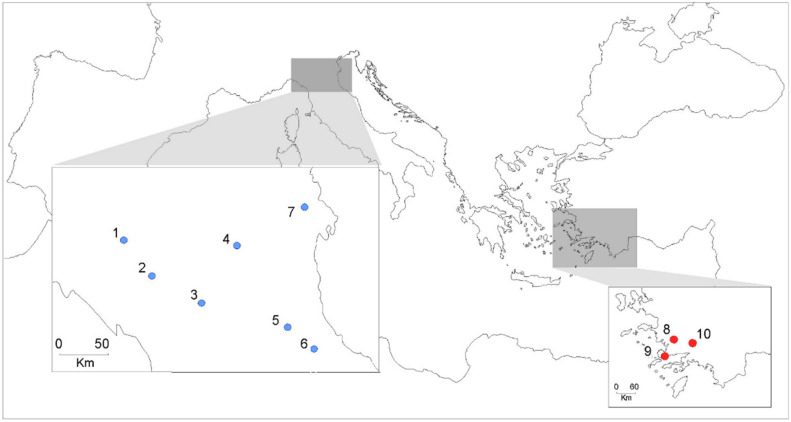
Map showing the location of the *Culex pipiens* samples used in this study. Population name and geographical coordinates are shown in Table 1. Blue and red dots represent the sampling sites from Italy and Turkey, respectively.

Collection of *Cx. pipiens* individuals was performed in urban and peri-urban areas from 10 localities of Italy and Turkey (Figure 1), based on the previous detection of DFB resistance [10,11,12]. In each locality, larval samples were collected from at least five breeding sites to reduce the probability of collecting isofemale mosquitoes. Population name, geographical origin, samples size and year of collection are shown in Table 1. 

Mosquito larvae were identified morphologically to species [12,13] and stored in ethanol 90%. Since *Cx. quinquefasciatus* was recently documented in Turkey, the taxonomic status of all larvae analyzed from Turkey was verified also by molecular analysis using the acetylcholinesterase (ACE) rapid assay as described in Smith and Fonseca [14].

### 2.2. Laboratory Procedures 

DNA from individual mosquitoes was extracted by standard CTAB (cetyltrimethyl ammonium bromide) protocol [15]. A fragment of the *chs-1* gene that encompass the site 1043 of point mutations responsible for DFB resistance was amplified by Polymerase Chain reaction using the primers CHSseqF (5′-CCGCGTTCAAGATTGACAACTGG-3′) and CHSseqR (5′TCCAGTAGGGGTTCGTCAGG-3′) [9,10].

The PCR reaction was carried out in a 25 µL volume containing 5 ng of DNA, 0.2 μM of the forward and reverse primers, 12.5 μL of Q5^®^ Hot Start High-Fidelity 2X Master Mix (Vetro Scientifica, Roma, Italy) and water. Negative controls included all reagents, but DNA were also included in the reaction. PCR cycling procedure was 95 °C for 5 min, 30 cycles of 94 °C for 30 sec, 60 °C for 30 sec, 72 °C for 1 min, and a final extension of 72 °C for 10 min. Four microliters of the PCR products were run on 1% agarose, 0.5X TAE gel, and visualized by staining with Gelred (Sigma-Aldrich, Milan, Italy) to check for quality. PCR products were then sequenced by standard Sanger sequencing by Microsynth Inc., Balgach, Switzerland (https://www.microsynth.ch.html). All individuals were double strand sequenced.

### 2.3. Sequence Analyses

Sequences were checked using the software Chromas 2.6.5 (Technelysium, Helensvale, Australia) and aligned using ClustalX 2.149. Heterozygous positions were identified by double peaks in the electropherograms and were encoded using the IUPAC ambiguity code. All homozygous sequences were submitted to GenBank (accession codes: MZ494156- MZ494187) and used for the following analyses. The RDP, Geneconv, MaxChi, and Chimaera tests as implemented in the program RDP4 [16], were used to detect the possible occurrence of recombination. Polymorphisms of nucleotide sequences, gene, and nucleotide diversity were estimated using the software DNASP v. 6.0. [17]. The average uncorrected *p*-distance between chs-alleles was computed using Mega 7.051 [18].

The phylogenetic relationships among the chs-alleles were inferred by constructing a phylogenetic network [19]. The statistical parsimony algorithm [20], as implemented in the software TCS, was used, applying a 95% cut-off for the probability of a parsimonious connection.

## 3. Results

A total of 266 sequences of a 608 bp fragment of the *chs-1* gene encompassing the site 1043 were obtained: among them, 124 sequences were homozygous (i.e., without double peaks in any position) and were used for DNA polymorphism and phylogenetic analyses (Table 1).

DNA polymorphism analysis showed the occurrence of 32 chs-alleles (encoded as a1–a32) (Table 1), identified by 57 nucleotide substitutions in 61 polymorphic sites (57 parsimony informative). Mean gene diversity and nucleotide diversity estimates were, respectively: 0.945 (sd 0.004) and 0.0189 (sd 0.0036). The recombination analysis did not find any event within the sequences (all *p* values > 0.05). Average uncorrected *p*-distance between the chs-alleles was 0.0189 (standard error 0.0026).

**Figure 2:**
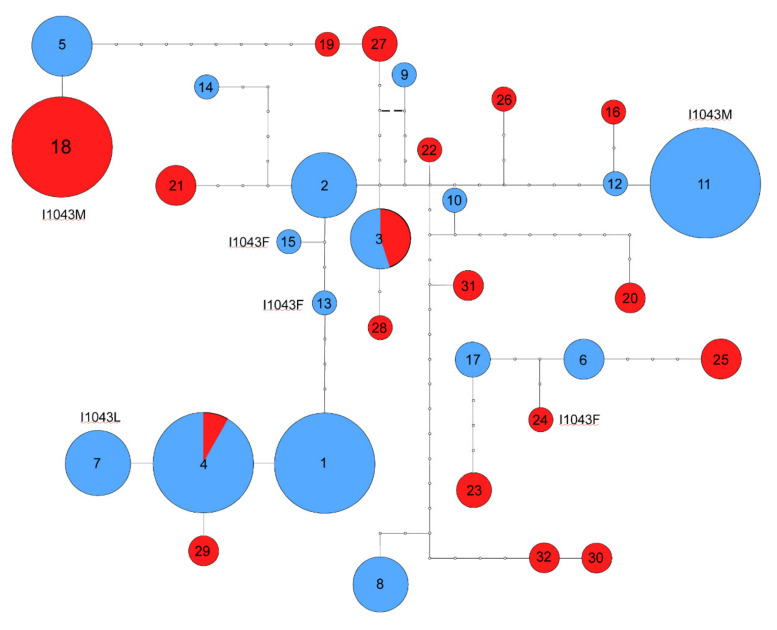
Statistical parsimony network showing the phylogenetic relationships among the 32 chs-alleles observed in the *Culex pipiens* populations. Alleles are shown as circles with sizes corresponding to their frequencies in the total sample. Alleles are encoded as in Table 1. Open dots are missing intermediate haplotypes. For each resistant allele, the name of the mutation is specified.

Six chs-alleles carrying DFB-resistant mutations were found (Table 1 and Table 2 and Figure 2). Among them, two alleles (a11 and a18) carried the I1043M mutation: the a11 allele was found in the Forlì and Cesena populations from Italy, while the a18 allele was found in the Turkish populations of Milas and Bodrum (uncorrected p-distance between them is 0.0339) (Supplementary Table S1). One allele carrying the I1043L mutation was found only in the Italian populations (a7). Three alleles carrying the I1043F mutation were found (a13, a15, and a24). The a13 and a15 alleles were found only in Italy, and they are closely related (uncorrected *p*-distance 0.0049); the a24 allele was found in Turkey. The uncorrected *p*-distances between the a13, a15 Italian alleles and the Turkish a24 allele were 0.0150 and 0.0167, respectively (Supplementary Table S1).

The phylogenetic relationships between the chs-alleles were reconstructed using Statistical Parsimony [19,20]. As shown by the network in Figure 2, no evidence of geographical structuring was found, as the alleles collected in Turkey were not more closely related among them than with the alleles collected in Italy.

The Italian alleles carrying the I1043M mutation (a11) originated from the susceptible allele a12 found in Forlì. The Italian alleles that carry the I1043L mutation (a7) originated from the susceptible allele a4 that occurs in six out of seven Italian populations (Table 1, Figure 2). The Italian alleles carrying the I1043F mutation (a13 and a15) originated from the susceptible allele a2 that was found in the Fiorenzuola and Parma populations (Table 1, Figure 2).

The Turkish allele carrying the I1043M mutation (a18) originated from the susceptible allele a5. This allele was found in the Italian population of Fiorenzuola, Parma, Bologna, and Forlì. Likewise, the a24 allele carrying the I1043F mutation is closely related to the susceptible allele a6 found in Italy in the Bologna and Mizzana populations and to the susceptible allele a17 found in the Italian population of Rovigo populations (Table 1, Figure 2).

## 4. Discussion

Three nonsynonymous substitutions in the 1043 codon of the *chs-1* gene (I1043M, I1043L, and I1043F) are involved in resistance to DFB insecticide in *Cx. pipiens* populations [10]. All three resistant alleles have been documented in Northern Italy [10], and both the I1043M and I1043L alleles were found in *Cx. pipiens* populations from Turkey [12]. Confirming previous findings, we found the I1043M, I1043L, and I1043F alleles in Northern Italy and the I1043M allele in Turkey. At the same time, we did not find the I1043L allele in the Turkish samples. Although updating the distribution and abundance of resistance mutations in the study areas is out this paper’s aim, the finding of the I1043F allele also in the Milas population is worthy of attention.

How many times a resistance mutation appears is a central issue in understanding the evolution of resistance and its subsequent spread upon different insecticide selection pressure. Here, by using a phylogenetic approach, we firstly investigated whether the I1043F, I1043M, and I1043L mutations originated independently from different susceptible alleles, or they came from mutations in a resistant allele. For example, the TTC mutation (I1043F phenotype) could have originated from the ATC-susceptible allele (I1043 phenotype) by one point mutation, from the CTC-resistant allele (I1043L phenotype) by one point mutation, or from the ATG-resistant allele (I1043M phenotype) by two point mutations. Our analyses strongly support the multiple origins of all three resistant alleles. The chs-alleles that carry the mutations I1043L, I1043M, and I1043F, indeed, differ by several nucleotide substitutions and are identical to different susceptible alleles, except for the associated resistant mutation or few additional nucleotide substitutions (Figure 2).

Secondly, we investigated whether the I1043M and I1043F alleles found in Northern Italy and Turkey evolved multiple times independently. Regarding the chs-alleles carrying the I1043M mutation, the phylogenetic analysis showed that the a11 allele from Italy is substantially different from the a18 allele from Turkey, which suggests a separate origin. Likewise, the chs-alleles that carry the I1043F mutation from Italy (a13 and 15) are closely related to each other but distantly related to the I1043F allele from Turkey (a24), again supporting an independent origin.

Regarding the geographic origin of the I1043M- and I1043F-resistant alleles, the differences observed in the flanking regions of the I1043M and I1043F mutations observed in Italy and Turkey would suggest an independent origin. Multiple evolutionary origins in separate geographic areas have been suggested in different species for point mutations at the insecticide target sites, such as the gamma-aminobutyric acid (GABA) receptors [21], the acetylcholinesterase [22], and the voltage-gated sodium channel (VGSC) [23,24]. However, because the *chs-1* region analyzed in *Cx. pipiens* showed no phylogeographic structure, future genetic populations studies using Single Nucleotide Polymorphims (SNPs) or microsatellite genetic markers are needed to shed light on this issue.

DFB resistance is of major concern for *Cx. pipiens* control, as DFB is one of the very few active ingredients available in Europe against mosquito larvae [1,11,25]. To date, the Northern Italy and Southwestern Turkey are the only geographic regions where multiple resistant alleles have been documented since the first detection of DFB resistance in 2015 in Italy [9,12]. Here, we showed that the three resistant mutations most likely originated from different susceptible alleles, and that the I1043M and I1043F alleles from Italy and Turkey evolved multiple times independently upon selective pressure from DFB applications. These findings, along with the first report of the I1043F mutation in Turkey, highlight the need for further monitoring activities in the Mediterranean region, where DFB is used against mosquito vectors.

## Figures and Tables

**Table 1:** Locations, year of collection of the *Culex pipiens* samples analyzed in this study and number of homozygous sequences found in each locality. In brackets how many times a chs-allele was found in the population is shown. The chs-alleles that carry the DFB-resistant mutations are shown in bold: a7 (I1043L mutation); a11 and a18 (I1043M mutation); a13, a15, and a24 (I1043F mutation).

Country	Code. Locality	No Larvae Analyzed	No. Homozygous Genotypes	Lat.	Long.	Year	chs-alleles
Italy	1. Fiorenzuola	28	12	44,9241667 N	9,9130555 E	2017	a1(6), a2(10), a3(2), a4(2), a5(4)
	2. Parma	26	11	44,768382 N	10,319429 E	2017	a1(4), a2(6), a3(4), a4(4), a5(4)
	3. Bologna	25	11	44,533022 N	11,344763 E	2020	a4(6), a5(2), a6(2), **a7**(6), a8(6)
	4. Mizzana	27	12	44,851931 N	11,588654 E	2017	a1(8), a4(4), a6(6), a8(2), a9(2), a10(2)
	5. Forlì	25	13	44,2109124 N	12,0552449 E	2020	a5(4), **a11**(16), a12(2), **a13**(2), a14(2)
	6 Cesena	25	12	44,1425853 N	12,2596991 E	2020	a4(2), **a7**(8), **a11**(10), **a15**(2), a16(2)
	7. Rovigo	24	10	45,069454 N	11,790022 E	2018	a1(4), a3(2), a4(2), **a7**(2), a8(4), a17(6)
Turkey	8. Milas	30	16	36,796029 N	28,796965 E	2016	a3(2), **a18**(10), a19(2), a20(2), a21(4), a22(2), a23(6), **a24**(2), a25(2)
	9. Bodrum	28	15	37,058288 N	27,233579 E	2016	**a18**(12), a21(4), a26(2), a27(6), a28(2), a29(2), a30(2)
	10. Menteşe	28	12	37,253156 N	28,244305 E	2016	a3(4), a4(2), a20(2), a25(6), a29(2), a31(4), a32(4)

**Table 2:** Variable sites within the *chs*-1 alleles that carry the DFB-resistant mutations. Numbering starts from the first nucleotide of the 608 bp amplified sequence. In yellow, the DFB mutations in the codon I1043 (ATC) are shown: I1043L (CTC); I1043M (ATG); I1043F (TTC).

Allele			Variable sites																																																								
			2 4	2 8	3 4	4 6	5 2	6 4	9 1	1 1 2	1 2 4	1 3 9	1 4 8	1 5 4	1 7 5	1 9 0	1 9 9	2 2 3	2 3 8	2 4 1	2 7 1	2 8 9	2 9 0	2 9 2	2 9 5	3 1 1	3 1 7	3 1 9	3 3 1	3 5 8	3 6 1	3 6 7	3 7 3	3 8 8	3 9 1	4 0 9	4 1 8	4 2 1	4 2 7	4 3 9	4 5 4	4 6 9	4 7 2	5 1 1	5 1 7	5 2 0	5 2 3	5 3 2	5 5 6	5 5 9	5 6 0	5 6 5	5 7 4	5 7 7	5 8 0	5 8 1	6 0 0	6 0 1	6 0 4
a1			T	T	G	T	G	G	G	G	C	C	C	A	C	G	G	C	C	T	C	T	A	C	G	T	T	G	C	T	C	T	G	G	A	G	A	A	C	G	C	A	A	A	C	C	C	C	G	C	T	C	C	G	G	T	T	G	C
a2			-	-	-	-	-	-	-	-	-	-	-	-	-	-	-	T	-	-	-	C	-	-	A	-	C	-	-	-	-	-	-	-	-	-	-	-	T	-	-	-	-	-	-	-	-	-	-	-	-	-	-	-	-	-	-	-	-
a3			-	-	-	-	-	-	-	-	-	-	-	G	-	C	-	T	-	-	-	C	-	-	A	-	C	-	-	-	-	-	-	-	-	-	-	-	T	-	-	-	-	-	-	-	-	-	-	-	-	-	-	-	-	-	-	-	-
a4			-	-	-	-	-	-	-	-	T	-	-	-	-	-	-	-	-	-	-	-	-	-	-	-	-	-	-	-	-	-	-	-	-	-	-	-	-	-	-	-	-	-	-	-	-	-	-	-	-	-	-	-	-	-	-	-	-
a5			-	-	-	-	-	-	A	-	-	-	-	-	-	A	-	T	-	C	T	C	-	-	-	-	C	-	-	-	-	-	-	-	-	-	-	-	-	-	-	C	T	-	-	-	T	T	-	-	A	-	-	-	A	C	-	C	-
a6			-	-	-	-	-	-	-	-	-	-	-	-	-	-	-	T	-	-	-	C	-	-	-	-	C	-	T	C	-	C	-	A	-	A	-	-	-	-	-	-	-	-	-	-	-	-	-	-	-	-	-	-	-	-	-	-	-
**a7**			-	-	-	-	-	-	-	-	T	-	-	-	-	-	-	-	-	-	-	-	C	-	-	-	-	-	-	-	-	-	-	-	-	-	-	-	-	-	-	-	-	-	-	-	-	-	-	-	-	-	-	-	-	-	-	-	-
a8			C	C	-	-	A	-	-	T	-	T	-	-	-	C	A	T	-	-	-	C	-	-	-	-	C	-	-	-	A	C	-	A	-	A	-	-	-	-	T	-	-	-	-	-	-	-	-	-	-	-	-	-	-	-	-	-	-
a9			-	-	-	-	-	-	-	-	-	-	-	-	-	C	-	T	-	-	-	C	-	-	-	-	C	-	-	-	-	-	-	-	-	-	-	-	-	-	-	-	-	-	-	-	T	-	A	-	A	-	-	-	-	C	-	-	-
a10			-	-	-	-	-	-	-	-	T	-	-	-	-	C	-	T	-	-	-	C	-	-	-	-	C	-	-	-	-	-	-	-	G	A	-	-	-	A	-	-	-	-	-	-	-	-	-	-	-	-	-	-	-	-	-	-	-
**a11**			C	C	-	-	-	A	-	-	-	-	-	-	T	A	-	T	-	-	-	C	-	G	A	-	C	-	-	-	A	-	-	-	-	A	-	-	-	-	-	-	-	-	-	-	-	-	-	-	-	-	-	-	-	-	-	-	-
a12			C	C	-	-	-	A	-	-	-	-	-	-	T	A	-	T	-	-	-	C	-	-	A	-	C	-	-	-	A	-	-	-	-	A	-	-	-	-	-	-	-	-	-	-	-	-	-	-	-	-	-	-	-	-	-	-	-
**a13**			-	-	-	-	-	-	-	-	-	-	-	-	-	-	-	T	-	-	-	-	T	-	A	-	C	-	-	-	-	-	-	-	-	-	-	-	-	-	-	-	-	-	-	-	-	-	-	-	-	-	-	-	-	-	-	-	-
a14			-	-	-	-	-	-	-	-	-	-	-	-	-	-	-	T	-	-	-	C	-	-	A	-	C	-	-	-	-	-	-	-	-	-	G	-	T	-	C	-	-	G	-	T	-	-	-	-	A	-	-	A	-	-	-	-	T
**a15**			-	-	-	-	-	-	-	-	-	-	T	-	-	-	-	T	-	-	-	C	T	-	A	-	C	-	-	-	-	-	-	-	-	-	-	-	T	-	-	-	-	-	-	-	-	-	-	-	-	-	-	-	-	-	-	-	-
a16			C	C	-	-	-	A	-	-	-	-	-	-	T	A	-	T	-	-	-	C	-	-	A	-	C	-	-	-	A	-	-	-	-	A	-	-	-	A	-	-	-	-	-	-	-	-	-	-	-	-	-	-	-	C	-	C	-
a17			-	-	-	-	-	-	-	-	-	-	-	-	-	C	-	T	-	-	-	C	-	-	-	-	C	-	-	C	-	C	-	A	-	A	-	-	-	-	T	-	-	-	-	-	-	-	-	-	-	-	-	-	-	-	-	-	-
a18			-	-	-	-	-	-	A	-	-	-	-	-	-	A	-	T	-	C	T	C	-	G	-	-	C	-	-	C	-	-	-	-	-	-	-	-	-	-	C	C	T	-	-	-	T	T	-	-	A	-	-	-	A	C	-	C	-
a19			-	-	-	-	-	-	-	-	-	-	-	-	-	C	-	T	-	-	-	C	-	-	A	-	C	-	-	-	-	-	-	-	-	-		-	T	-	-	-	-	-	-	-	T	T	-	-	A	-	-	-	A	C	-	C	-
a20			-	-	-	C	-	-	-	-	-	-	-	G	-	-	-	T	A	-	-	C	-	-	-	-	C	-	-	-	-	-	-	-	G	A	-	-	-	A	-	-	-	-	-	-	-	-	-	-	A	T	-	-	A	C	-	C	-
a21			-	-	-	-	-	-	-	-	-	-	-	-	-	A	-	T	-	-	-	C	-	-	A	-	C	-	-	-	-	-	-	-	-	-	-	G	T	-	-	-	-	-	-	-	-	-	-	T	A	-	-	-	-	-	-	-	-
a22			-	-	-	-	-	-	-	-	-	-	-	-	-	C	-	T	-	-	-	C	-	-	A	-	C	-	-	-	-	-	-	-	-	A	-	-	-	-	-	-	-	-	-	-	-	-	-	-	-	-	-	-	-	-	A	-	-
a23			-	-	-	-	-	-	-	-	-	-	-	G	-	C	-	T	-	-	-	C	-	T	-	-	C	-	-	C	-	C	T	A	-	A	-	-	-	-	T	-	-	-	-	-	-	-	-	-	-	-	-	-	-	-	A	-	-
**a24**			-	-	A	-	-	-	-	-	-	-	-	-	-	C	-	T	-	-	-	C	T	-	-	-	C	-	T	C	-	C	-	A	-	A	-	-	-	-	-	-	-	-	-	-	-	-	-	-	-	-	-	-	-	-	-	-	-
a25			C	C	-	-	-	-	-	-	T	-	-	-	-	-	-	T	-	-	T	C	-	-	-	-	C	-	T	C	-	C	-	A	-	A	-	-	-	-	-	-	-	-	-	-	-	-	-	-	-	-	-	-	-	-	-	-	-
a26			-	-	-	-	-	-	-	A	-	-	-	-	-	A	-	T	-	-	-	C	-	-	A	C	C	A	-	-	A	-	-	-	-	A	-	-	-	A	-	-	-	-	-	-	-	-	-	-	-	-	-	-	-	-	-	-	-
a27			-	-	-	-	-	-	-	-	-	-	-	-	-	C	-	T	-	-	-	C	-	-	A	-	C	-	-	-	-	-	-	-	-	-	-	-	T	-	-	-	-	-	-	-	T	T	-	-	A	-	-	-	A	C	-	-	-
a28			-	-	-	-	-	-	-	-	-	-	-	G	-	C	-	T	-	-	-	C	-	-	A	-	C	-	-	-	-	-	-	-	-	-	-	-	T	-	-	-	-	-	T	-	-	C	-	-	-	-	A	-	-	-	-	-	-
a29			-	-	-	-	-	-	-	-	T	-	-	-	-	-	-	-	-	-	-	C	-	-	-	-	-	-	-	-	-	-	-	-	-	-	-	-	-	-	-	-	-	-	-	-	-	-	-	-	-	-	-	-	-	-	-	-	-
a30			C	C	-	-	A	-	-	-	-	T	-	-	-	A	-	T	-	-	-	C	-	-	-	-	C	-	-	-	A	-	-	-	-	A	-	-	-	A	-	-	-	-	-	-	-	-	-	-	A	-	-	A	-	-	-	-	-
a31			-	-	A	-	-	-	-	-	-	-	-	-	-	C	-	T	-	-	-	C	-	-	-	-	C	-	-	-	-	-	-	-	G	A	-	-	-	-	T	-	-	-	-	-	-	-	-	-	-	-	-	-	-	-	-	-	-
a32			C	C	-	-	A	-	-	-	-	T	-	-	-	A	-	T	-	T	-	C	-	-	-	-	C	-	-	-	A	-	-	-	-	A	-	-	-	A	-	-	-	-	-	-	-	-	-	-	-	-	-	A	-	-	-	-	-

## Data Availability

Data can be found within the article and the Supplementary Materials.

## Supplementary Materials

The following are available online at https://www.mdpi.com/article/10.3390/insects12080676/s1, Table S1: Average uncorrected *p*-distance between the chs-alleles found in *Culex pipiens* populations.
