# Supplementary material for: Evolution of Adaptive Variation in the Mosquito Culex pipiens: Multiple Independent Origins of Insecticide Resistance Mutations
Source: Insects. 2021 Jul 27;12(8):676. doi: 10.3390/insects12080676 (PMC8396673; doi:10.3390/insects12080676)
Supplement: Supplementary file 1 [file insects-12-00676-s001.zip › insects-1312470-supplementary.pdf]

Supplementary Table S1. Average uncorrected p-distance between the chs-alleles found in *Culex pipiens* populations. The alleles are numbered from a1 to 32 as shown in Figure 2. In bold the chs-alleles carrying the DFB mutations are shown: a7 (I1043L mutation); a11 and a18 (I1043M mutation); a13, a15, and a24 (I1043F mutation).

	a1	a2	a3	a4	a5	a6	**a7**	a8	a9	a10	**a11**	a12	**a13**	a14	**a15**	a16	a17	**a18**	a19	a20	a21	a22	a23	**a24**	a25	a26	a27	A28	a29	a30	a31	a32
a1	*****																															
a2	0,0083	*****																														
a3	0,0116	0,0033	*****																													
a4	0,0016	0,0099	0,0133	*****																												
a5	0,0269	0,0252	0,0270	0,0286	*****																											
a6	0,0133	0,0116	0,0150	0,0149	0,0269	*****																										
**a7**	0,0033	0,0116	0,0150	0,0016	0,0304	0,0166	*****																									
a8	0,0252	0,0235	0,0235	0,0269	0,0409	0,0184	0,0286	*****																								
a9	0,0150	0,0100	0,0099	0,0167	0,0201	0,0184	0,0184	0,0269	*****																							
a10	0,0133	0,0116	0,0116	0,0116	0,0287	0,0133	0,0133	0,0218	0,0150	*****																						
**a11**	0,0218	0,0167	0,0184	0,0234	0,0356	0,0218	0,0252	0,0218	0,0218	0,0167	*****																					
a12	0,0200	0,0150	0,0167	0,0217	0,0339	0,0200	0,0234	0,0201	0,0201	0,0150	0,0016	*****																				
**a13**	0,0066	0,0050	0,0083	0,0083	0,0270	0,0133	0,0083	0,0252	0,0116	0,0133	0,0184	0,0167	*****																			
a14	0,0183	0,0100	0,0133	0,0200	0,0320	0,0217	0,0217	0,0338	0,0167	0,0218	0,0269	0,0252	0,0150	*****																		
**a15**	0,0116	0,0033	0,0066	0,0133	0,0287	0,0150	0,0133	0,0269	0,0133	0,0150	0,0201	0,0184	0,0049	0,0133	*****																	
a16	0,0234	0,0184	0,0201	0,0251	0,0304	0,0234	0,0269	0,0235	0,0201	0,0184	0,0050	0,0033	0,0201	0,0286	0,0218	*****																
a17	0,0150	0,0133	0,0133	0,0166	0,0270	0,0050	0,0184	0,0133	0,0167	0,0116	0,0218	0,0201	0,0150	0,0235	0,0167	0,0235	*****															
**a18**	0,0287	0,0270	0,0287	0,0304	0,0016	0,0287	0,0321	0,0427	0,0218	0,0304	0,0339	0,0356	0,0287	0,0338	0,0304	0,0321	0,0287	*****														
a19	0,0201	0,0116	0,0116	0,0218	0,0150	0,0235	0,0235	0,0321	0,0083	0,0201	0,0270	0,0252	0,0167	0,0184	0,0150	0,0218	0,0217	0,0167	*****													
a20	0,0235	0,0218	0,0218	0,0252	0,0269	0,0235	0,0269	0,0357	0,0218	0,0167	0,0287	0,0269	0,0235	0,0286	0,0252	0,0235	0,0252	0,0287	0,0201	*****												
a21	0,0150	0,0066	0,0083	0,0166	0,0252	0,0183	0,0184	0,0287	0,0116	0,0167	0,0201	0,0184	0,0116	0,0133	0,0100	0,0218	0,0184	0,0269	0,0133	0,0252	*****											
a22	0,0116	0,0066	0,0066	0,0133	0,0270	0,0116	0,0150	0,0201	0,0100	0,0083	0,0150	0,0133	0,0083	0,0167	0,0100	0,0167	0,0099	0,0288	0,0150	0,0219	0,0117	*****										
a23	0,0218	0,0201	0,0167	0,0235	0,0339	0,0116	0,0252	0,0201	0,0235	0,0184	0,0270	0,0269	0,0218	0,0304	0,0235	0,0304	0,0066	0,0340	0,0286	0,0287	0,0252	0,0133	*****									
**a24**	0,0184	0,0167	0,0167	0,0200	0,0304	0,0050	0,0200	0,0201	0,0201	0,0150	0,0252	0,0235	0,0150	0,0269	0,0167	0,0269	0,0066	0,0322	0,0252	0,0287	0,0218	0,0133	0,0133	*****								
a25	0,0200	0,0183	0,0217	0,0183	0,0304	0,0066	0,0200	0,0184	0,0251	0,0166	0,0218	0,0200	0,0200	0,0285	0,0217	0,0234	0,0116	0,0321	0,0303	0,0303	0,0251	0,0184	0,0184	0,0116	*****							
a26	0,0184	0,0133	0,0150	0,0200	0,0321	0,0184	0,0218	0,0235	0,0184	0,0133	0,0133	0,0116	0,0150	0,0235	0,0167	0,0150	0,0184	0,0339	0,0235	0,0252	0,0167	0,0117	0,0252	0,0218	0,0251	*****						
a27	0,0184	0,0100	0,0099	0,0200	0,0167	0,0217	0,0218	0,0303	0,0066	0,0183	0,0252	0,0235	0,0150	0,0167	0,0133	0,0235	0,0200	0,0184	0,0016	0,0218	0,0116	0,0133	0,0269	0,0234	0,0285	0,0218	*****					
a28	0,0150	0,0066	0,0033	0,0167	0,0304	0,0184	0,0184	0,0269	0,0133	0,0150	0,0218	0,0201	0,0116	0,0167	0,0100	0,0235	0,0167	0,0322	0,0150	0,0253	0,0117	0,0100	0,0201	0,0201	0,0251	0,0184	0,0133	*****				
a29	0,0033	0,0083	0,0116	0,0016	0,0269	0,0133	0,0033	0,0252	0,0150	0,0100	0,0218	0,0200	0,0099	0,0183	0,0116	0,0234	0,0150	0,0287	0,0201	0,0235	0,0150	0,0116	0,0218	0,0184	0,0166	0,0184	0,0184	0,0150	*****			
a30	0,0218	0,0201	0,0218	0,0234	0,0321	0,0218	0,0252	0,0150	0,0218	0,0167	0,0133	0,0116	0,0218	0,0234	0,0235	0,0150	0,0218	0,0339	0,0269	0,0252	0,0200	0,0184	0,0287	0,0252	0,0218	0,0167	0,0252	0,0252	0,0218	*****		
a31	0,0133	0,0116	0,0116	0,0150	0,0287	0,0133	0,0167	0,0184	0,0150	0,0066	0,0201	0,0184	0,0133	0,0218	0,0150	0,0218	0,0083	0,0304	0,0201	0,0201	0,0167	0,0083	0,0150	0,0116	0,0200	0,0167	0,0183	0,0150	0,0133	0,0201	*****	
a32	0,0200	0,0184	0,0201	0,0217	0,0339	0,0200	0,0234	0,0133	0,0235	0,0150	0,0116	0,0099	0,0201	0,0252	0,0218	0,0133	0,0201	0,0356	0,0287	0,0269	0,0218	0,0167	0,0269	0,0235	0,0200	0,0150	0,0269	0,0235	0,0200	0,0016	0,0184	*****
